# Taxonomic revision of *Aegista subchinensis* (Möllendorff, 1884) (Stylommatophora, Bradybaenidae) and a description of a new species of *Aegista* from eastern Taiwan based on multilocus phylogeny and comparative morphology

**DOI:** 10.3897/zookeys.445.7778

**Published:** 2014-10-13

**Authors:** Chih-Wei Huang, Yen-Chen Lee, Si-Min Lin, Wen-Lung Wu

**Affiliations:** 1 Department of Life Science, National Taiwan Normal University, 88 Ting-Chow Rd, Sec 4, Taipei, 11677 Taiwan; 2 Biodiversity Research Center, Academia Sinica, 128 Academia Road Sec. 2, Nankang Taipei 11529 Taiwan

**Keywords:** Stylommatophora, Helicoidea, Southern Ryukyu Islands, Yaeyama Islands, new species

## Abstract

*Aegista subchinensis* (Möllendorff, 1884) is a widely distributed land snail species with morphological variation and endemic to Taiwan. Three genetic markers (partial sequence of the mitochondrial cytochrome c oxidase subunit I [COI], the 16S rDNA and the nuclear internal transcribed spacer 2 [ITS2]) were analysed to infer phylogenetic relationships and genetic divergence of closely related species of the genus *Aegista*, *Aegista vermis* (Reeve, 1852) and *Aegista oculus* (Pfeiffer, 1850). A new species from *Aegista subchinensis* has been recognized on the basis of phylogenetic and morphological evidences. The nominal new species, *Aegista diversifamilia* **sp. n.** is distinguished from *Aegista subchinensis* (Möllendorff, 1884) by its larger shell size, aperture and apex angle; wider umbilicus and flatter shell shape. The northernmost distribution of *Aegista diversifamilia* **sp. n.** is limited by the Lanyang River, which is presumed to mark the geographic barrier between *Aegista diversifamilia* **sp. n.** and *Aegista subchinensis*.

## Introduction

Traditional morphology-based taxonomy provides a window to explore biodiversity and evolutionary history. The application of molecular genetic markers opens new avenues to discover biodiversity. In recent years, it was found that the species richness of land snails bearing comparably few morphological characteristics and exhibiting limited abilities of dispersal had been underestimated once molecular tools were applied (Hirano et al. 2014, Nantarat et al. 2014, Nishi and Sota 2007, Prevot et al. 2013, Wu et al. 2008). In contrast, taxonomic revision based merely on a single molecular locus may lead to an overestimation of number of taxa. Integrative taxonomy was therefore proposed to integrate multiple independent lines of evidence for objective taxonomic treatment (Dayrat 2005, Padial et al. 2010, Schlick-Steiner et al. 2010).

Taiwan is a continental island that was formed through the collision of the Philippine Sea fig and the Eurasian fig. This collision uplifted the Central Mountain Range (CMR), forming a major physical barrier for animals inhabit lowland areas. The CMR has contributed to evolutionary divergences between organisms on either side of the CMR both on interspecific and intraspecific levels (Huang and Lin 2010, Jang-Liaw et al. 2008, Kuo et al. 2014, Lin et al. 2012, Wang et al. 2007, Yu et al. 2014). *Aegista subchinensis* (Möllendorff, 1884) is one of the most widely distributed species of the genus *Aegista* in Taiwan and is commonly found in lowland hardwood forests near the CMR (Hsieh 2003, Hsieh et al. 2006, Hsieh et al. 2013, Lee and Chen 2003, Lee and Wu 2004). The morphological differences observed between western and eastern populations indicate that the evolutionary diversification of this species complex may be underestimated and requiring further investigation (Lee and Chen 2003, Lee and Wu 2004). Based on multilocus sequence analyses and comparative morphology, we demonstrate that *Aegista* snails from eastern Taiwan, originally identified as *Aegista subchinensis*, represent a new species which is herein described as *Aegista diversifamilia* sp. n.

## Materials and methods

### Sample collection and molecular techniques

Live snails identified as *Aegista subchinensis* were collected from ten localities in Taiwan. Similar species, *Aegista vermis* (Reeve, 1852) of Ishigaki Island and *Aegista oculus* (Pfeiffer, 1850) of Miyako Island, were collected from two and four localities, respectively, on the southern Ryukyu Islands. Four congenic species, *Aegista mackensii* (Adams & Reeve, 1850), *Aegista granti* (Pfeiffer, 1865), *Aegista inrinensis* (Pilsbry & Hirase, 1905), and *Aegista shermani* (Pfeiffer, 1865), distributed in Taiwan were used as outgroups to root the phylogenetic tree. Global positioning system (GPS) coordinates of sampling sites (including latitude, longitude and altitude) were recorded using Garmin GPSmap 60CSx with an uncertainty of less than 10 metres (Figure 2 and Table 1). Vouchers and type specimens of *Aegista subchinensis* and *Aegista diversifamilia* sp. n. were deposited in the National Museum of Natural Science, Taiwan (NMNS, NMNS-7276) and the Natural History Museum, United Kingdom (NHMUK 20140070–20140074). Snails were relaxed in water for at least 6 hours, quickly fixed by submersion in boiling water and then preserved in 95% ethanol. DNA was extracted from 10 mg of foot tissue using AxyPrep™ Multisource Genomic DNA Miniprep Kit (Axygen Bioscience, USA) following the manufacturer’s protocol. A partial sequence of mitochondrial cytochrome *c* oxidase subunit I (COI) was amplified using the LCO1490 and HCO2198 universal primers (Folmer et al. 1994). Partial 16S ribosomal DNA was amplified using the 16Sar and 16Sbr universal primers (Palumbi et al. 1991). Complete nuclear internal transcribed spacer 2 (ITS2) was amplified using the LSU1 and LSU3 primers (Wade et al. 2006). The PCR mixture was composed of 10 ng DNA temfig, 1 μM primers, 1X Taq DNA polymerase 2.0 Master mix kit (Ampliqon, Denmark) and water. The total volume of the PCR mixture was 20 μl. PCR was performed under the following conditions: initial denaturation at 94 °C for 1 min followed by 36 cycles of denaturing at 94 °C for 30 s, annealing at 48 °C or 52 °C for 30 s and a final extension at 72 °C for 30 s. Primer annealing temperature was 48 °C for COI and 52 °C for 16S and ITS2. The size of the PCR products was checked under ultraviolet light after gel electrophoresis. The PCR mixture was purified using Genomics Universal DNA Purification kit (Genomics BioSci and Tech, Taiwan). Sanger sequencing was performed on an ABI PRISM 3730 DNA Analyzer at Institute of Cellular and Organismic Biology, Academia Sinica.

**Table 1.** Sampling information and GenBank accession number of DNA sequences

	GPS coordinates			Sample size		GenBank accession number		
Sampling locality	Latitude	Longitude	Altitude	N_G_	N_M_	COI	16S	ITS2
*Aegista subchinensis*								
# Wulai, Taipei	NA.	NA.	NA.	1	0	AB852655	NA.	AB852922
1. Linmei Shipan Trail, Jiaoxi Twp., I-Lan Co., Taiwan	24°49'41.88"N	121°43'56.34"E	286	2	2	KJ574361	KJ574321–KJ574322	KJ574382–KJ574383
2. Houtong, Ruifang District, New Taipei City, Taiwan	25°05'10.8"N	121°49'44.5"E	105	0	3	NA.	NA.	NA.
3. Zhishanyan, Taipei City, Taiwan	25°06'10.8"N	121°31'47.0"E	53	3	5	KJ574358–KJ574360	KJ574318–KJ574320	KJ574380–KJ574381
4. Datieliao Trail, Daxi Twp., Taoyuan City, Taiwan	24°50'59.22"N	121°18'46.20"E	433	0	1	NA.	NA.	NA.
5. Shimen Reservoir 1, Daxi Twp., Taoyuan City, Taiwan	24°49'08.58"N	121°16'27.72"E	323	0	5	NA.	NA.	NA.
6. Shimen Reservoir 2, Daxi Twp., Taoyuan City, Taiwan	24°48'57.24"N	121°15'09.90"E	198	0	1	NA.	NA.	NA.
7. Frog Rock, Jianshi Twp., Hsinchu Co., Taiwan	24°41'12.3"N	121°13'43.2"E	468	0	5	NA.	NA.	NA.
8. Fuxing Coal Mine, Jianshi Twp., Hsinchu Co., Taiwan	24°40'58.3"N	121°14'01.3"E	512	0	1	NA.	NA.	NA.
9. Jinping, Jianshi Twp., Hsinchu Co., Taiwan	24°40'41.5"N	121°15'15.5"E	638	0	1	NA.	NA.	NA.
10. Shishan Trail, Nanzhuang Twp., Miaoli Co., Taiwan	24°38'33.0"N	121°00'30.6"E	344	0	5	NA.	NA.	NA.
11. Fengmei, Nanzhuang Twp., Miaoli Co., Taiwan	24°32'44.8"N	121°01'41.7"E	695	0	2	NA.	NA.	NA.
12. Dacaopai, Sanyi Twp., Miaoli Co., Taiwan	24°22'29.10"N	120°47'52.40"E	523	0	3	NA.	NA.	NA.
13. Wu Shi Branch School, Heping District, Taichung City, Taiwan	24°17'34.8"N	120°56'07.8"E	650	0	1	NA.	NA.	NA.
14. Wushikeng, Heping District, Taichung City, Taiwan	24°12'46.49"N	120°56''44.16"E	894	2	0	KJ574364–KJ574365	NA.	NA.
15. Huanshan, Heping District, Taichung City, Taiwan	24°19'11.27"N	121°17'18.33"E	1560	2	1	KJ574362–KJ574363	KJ574323	KJ574384
*Aegista diversifamilia* sp. n.								
16. Anpingkeng, Dongshan Twp., I-Lan Co., Taiwan	24°36'52.5"N	121°46'38.1"E	70	4	5	KJ574339–KJ574342	KJ574299–KJ574302	KJ574385
17. Wushibi, Su'ao Twp., I-Lan Co., Taiwan	24°29'13.5"N	121°50'02.9"E	382	1	0	KJ574343	KJ574303	KJ574386
18. Chaoyang Trail, Nan'ao Twp., I-Lan Co., Taiwan	24°27'35.9"N	121°48'53.9"E	42	0	2	NA.	NA.	NA.
19. Heren 1, Xiulin Twp., Hualien Co., Taiwan	24°14'49.1"N	121°43'06.4"E	36	0	1	NA.	NA.	NA.
20. Heren 2, Xiulin Twp., Hualien Co., Taiwan	24°14'54.8"N	121°42'51.4"E	55	0	7	NA.	NA.	NA.
21. Heren Trail, Xiulin Twp., Hualien Co., Taiwan	24°13'58.5"N	121°42'27.73"E	50	5	1	KJ574344–KJ574348	KJ574304–KJ574308	KJ574387–KJ574390
22. Jinwen Tunnel, Xiulin Twp., Hualien Co., Taiwan	24°12'28.7"N	121°40'23.5"E	128	0	8	NA.	NA.	NA.
23. Northern Chongde Tunnel, Xiulin Twp., Hualien Co., Taiwan	24°11'31.08"N	121°39'41.01"E	62	2	2	KJ574349–KJ574350	KJ574309–KJ574310	NA.
24. Southern Chongde Tunnel, Xiulin Twp., Hualien Co., Taiwan	24°11'22.0"N	121°39'36.8"E	56	3	5	KJ574351–KJ574352	KJ574311–KJ574313	KJ574394–KJ574396
25. Sanjianwu, Xiulin Twp., Hualien Co., Taiwan	24°10'55.3"N	121°37'34.3"E	165	0	6	NA.	NA.	NA.
26. Taroko Service Center, Xiulin Twp., Hualien Co., Taiwan	24°09'31.9"N	121°37'20.7"E	100	0	6	NA.	NA.	NA.
27. Badagang, Xiulin Twp., Hualien Co., Taiwan	24°10'36.8"N	121°33'43.6"E	421	5	0	KJ574353–KJ574357	KJ574314–KJ574317	KJ574391–KJ574393
*Aegista oculus*								
# Miyako Island, Japan	NA.	NA.	NA.	1	0	AB852642	NA.	AB852909
28. Shimozaki, Miyako Island, Japan	24°50'03.78"N	125°16'50.58"E	32	3	0	KJ574328	KJ574281–KJ574282	KJ574370
29. Hirara 1, Miyako Island, Japan	24°48'03.12"N	125°18'58.86"E	42	1	0	NA.	KJ574283	KJ574372
30. Hirara 2, Miyako Island, Japan	24°47'58.50"N	125°19'02.94"E	44	3	0	KJ574329	KJ574284–KJ574286	KJ574371, KJ574373
31. Shimozato, Miyako Island, Japan	24°47'15.24"N	125°17'11.10"E	44	6	0	KJ574330–KJ574335	KJ574287–KJ574291	KJ574374–KJ574375
*Aegista vermis*								
# IriomoteIsland, Japan	NA.	NA.	NA.	1	0	AB852660	NA.	AB852927
32. Tozato, Ishigaki Island, Japan	24°27'18.6"N	124°14'17.5"E	94	1	0	NA.	KJ574292	KJ574376
33. Fukai, Ishigaki Island, Japan	24°26'59.28"N	124°12'04.98"E	62	6	0	KJ574336–KJ574338	KJ574293–KJ574298	KJ574377–KJ574379
*Aegista caerulea*								
# Ishigaki Island, Japan	NA.	NA.	NA.	1	0	AB852626	NA.	AB852893
**Outgroups**								
*Aegista granti*								
34. Fuyang Park, Taipei City, Taiwan	25°0'56.66"N	121°33'26.82"E	32	1	0	KJ574368	KJ574326	KJ574398
*Aegista inrinensis*								
35. Neiwan, Hengshan Twp., Hsinchu Co., Taiwan	24°42'18.2"N	121°10'58.7"E	268	1	0	KJ574367	KJ574325	KJ574399
*Aegista shermani*								
36. Lanren Rd., Manzhou Twp., Pingtung Co., Taiwan	22°02'25.8"N	120°50'58.8"E	48	1	0	KJ574366	KJ574324	KJ574397
*Aegista mackensii*								
37. Gueishan Island, Taiwan	24°50'35.9"N	121°56'52.6"E	157	1	0	KJ574369	KJ574327	KJ574400

N_G_: Number of specimen for Genetic analyses; N_M_: Number of specimen for Morphological analyses; #: sequence obtained from Hirano et al. (2014); NA.: not available.

### Phylogenetic reconstruction

Sequences were visually checked using Bioedit version 7.2.5 (Hall 1999) and deposited in GenBank (KJ574281–KJ574400, Table 1). The sequence of ingroups made available by Hirano et al. (2014) were incorporated into the phylogenetic reconstruction (Table 1). Sequences were aligned by MAFFT online version 7 (Katoh and Standley 2013) using default settings. Partitionfinder version 1.1.1 (Lanfear et al. 2012) was used to identify the best partition model for the protein-coding gene COI. The best-fit substitution model for each gene was evaluated via jModelTest version 2.1.4 (Darriba et al. 2012, Guindon and Gascuel 2003). The model filtering threshold of heuristic was set to 1.0 to increase the efficiency for evaluating 56 substitution models. The best-fit model was evaluated using BIC and DT criteria because their accuracy has been shown to outperform hLRT and AIC (Luo et al. 2010). Phylogenetic relationship was inferred from each gene and concatenated sequences using maximum likelihood and the Bayesian inference method. Phylogeny of maximum likelihood was inferred using PhyML 3.0 (Guindon et al. 2010) implemented in SeaView version 4.4.2 (Gouy et al. 2010). Parameters were set to empirical nucleotide frequencies with optimized invariable sites; the number of rate categories equalled four; tree searching operation as the best of nearest neighbour interchange (NNI) and subtree pruning and regrafting (SPR); BioNJ with optimized tree topology as the starting tree. Branch support confidences were estimated via traditional bootstrap with 100 replicates (Felsenstein 1985) and an fast and accurate alternative method, approximate likelihood-ratio test (aLRT) (Anisimova and Gascuel 2006). Phylogeny of Bayesian inference was performed using MrBayes version 3.2 (Ronquist et al. 2012). Two parallel runs of three heated chains and one cold chain were performed for 2–5 million generations and sampled every 1000 generations. Sampling was stopped when all chains reached stationarity and the runs converged (split frequencies < 0.01). The first 25% of sampling trees were discarded as burn-in and a 50% majority-rule consensus tree was computed. Branch support confidences were inferred from Bayesian posterior probability. Network is an alternative way to infer the phylogenetic relationship among haplotypes (Bandelt et al. 1999, Bapteste et al. 2013, Huson and Bryant 2006). Haplotype network was reconstructed by median-joining method (Bandelt et al. 1999) via NETWORK version 4.612 (available at http://www.fluxus-engineering.com/sharenet.htm). Interspecific and intraspecific genetic distances were calculated by using PAUP* version 4.0 (Swofford 2003) employing the best-fit substitution model but excluding sequences from Hirano et al. (2014).

### Morphological analyses

For genital morphological comparison, we dissected two samples of one adult snail each from the western (Zhishanyan, Taipei City) and the eastern (Heren 1, Xiulin Township, Hualien County) population, respectively (Table 1). Snails were relaxed in water for more than 6 hours, fixed by submersion in boiling water for 20 seconds, and preserved in 70% ethanol. Shell was crushed before dissection. All soft tissues were preserved in 70% ethanol. Terminology of genital morphology follows Gómez (2001). Images of genitalia were obtained via Canon PowerShot A650IS digital camera. QPcard 201 was included in each image for colour and scale correction with PhotoImpact X3 (Corel Corp., Taipei, Taiwan). The following organs were measured in cm and scaled by their shell width: length of albumen gland (AG), hermaphroditic duct (HD), spermoviduct (SOD), free oviduct (FO), vagina (V), dart-sac of auxiliary copulatory organ (DS), penis (P), epiphallus (E), and epiphallus flagellum (EF). The following ratios were calculated: HD/AG, AG/SOD, HD/SOD, FO/V, EF/E, E/P, and P/V.

**Figure 1.**
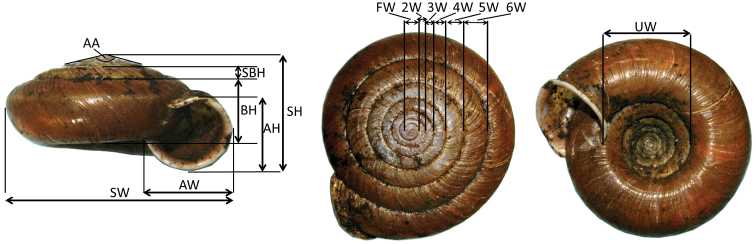
Morphometric measurement of shell size variation of *Aegista diversifamilia* sp. n. (shown in this figure) in top view, apertural view and umbilical view. **AA:** angle of apex; **AH:** aperture height; **AW:** aperture width; **BH:** body whorl height; **FW:** first whorl width; **SBH:** secondary body whorl height; **SH:** shell height; **SW:** shell width; **UW:** umbilicus width; **2W–6W:** 2nd–6th whorl width.

Shell morphological comparisons were based on examination of 36 specimens of *Aegista subchinensis* from the west of the CMR and 43 specimens from the east (Table 1). Morphological differences between the western and the eastern *Aegista subchinensis* were analysed on the basis of measurements and shape outline coordinates. Shell images were obtained as mentioned above. The image’s background was removed using Clipping Magic (available at https://clippingmagic.com/). Images were converted into thin-fig splines (TPS) format using tpsUtil version 1.56 (Rohlf 2013). The Janssen’s method of counting the number of whorls was used (Janssen 2007). Fifteen characteristics were measured: the number of whorls; width of the shell (SW), aperture (AW), umbilicus (UW), first whorl (FW), and 2^nd^–6^th^ whorl (2W–6W); height of the shell (SH), aperture (AH), body whorl (BH), and secondary body whorl (SBH); and angle of apex (AA) (Figure 1). Measurements of length (in cm) and angle were conducted using tpsDig version 1.4 (Rohlf 2004) and ImageJ version 1.47 (Abramoff et al. 2004), respectively. The shell flatness and aperture shape were expressed by calculating ratios of shell height divided by shell width (SH/SW), aperture height divided by aperture width (AH/AW) and umbilicus width divided by shell width (UW/SW), and additional ratios were calculated: AW/SW, BH/SW, SBH/SW, FW/SW, AH/SH, SBH/SH, SBH/SH, SBH/BH, SH/UW, AW/UW, AH/UW, BH/UW, SBH/UW, 2W/3W, 3W/4W, 4W/5W, 5W/6W. Summary statistics and 95% confidence intervals of these characteristics were calculated for the western and the eastern population of *Aegista subchinensis*. Statistical differences of each characteristic were analysed using Welch’s *t* test or the Mann–Whitney *U* test when the distribution of characteristics did or did not follow a normal distribution, respectively. Multivariate analysis of variance (MANOVA) was conducted with the species as independent variables and the characteristics mentioned above as dependent variables. A Bonferroni correction was applied for *p*-values. All characteristics were log-transformed for principle component analysis (PCA) and discriminant analysis with Jackknifed classification via PAST version 3.01 (Hammer et al. 2001). Outline coordinates of each shell were digitalized using tpsDig for geometric morphometric analysis of shell shape. Outline coordinates were approximated with 16 control points via Morphomatica version 1.6 (Linhart et al. 2006). The outline coordinates of the western and the eastern *Aegista subchinensis* were used to calculate mean shell shape in Morphomatica. A relative warps PCA was conducted using PAST.

## Results and discussion

### Molecular phylogeny

**Figure 2.**
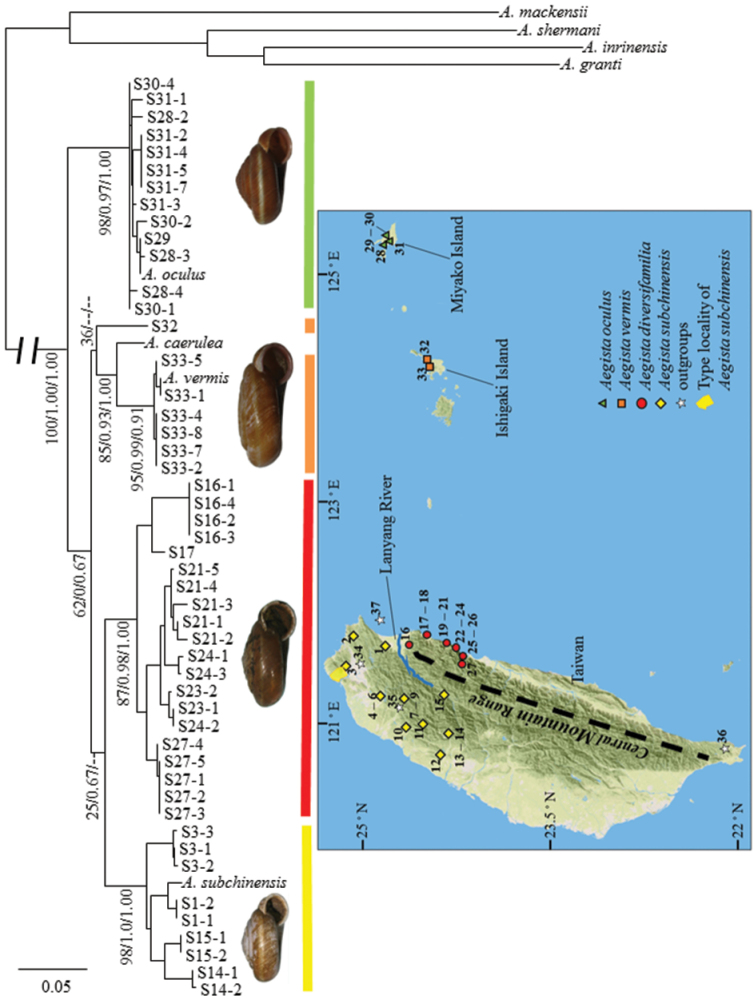
Maximum likelihood phylogeny and sampling sites of *Aegista* spp. Reconstructed phylogeny was based on concatenated sequences of mitochondrial COI, 16S and nuclear ITS2 genes. Branch support confidences of clades are shown in bootstrap, approximate likelihood-ratio test and Bayesian posterior probability, respectively. The log likelihood of maximum likelihood tree = -6584.1713. The numbered sampling sites are detailed in Table 1.

A total of 50 individuals were sequenced from the *Aegista* ingroup. The lengths of the COI, 16S and ITS2 after alignment were 655 bp, 280 bp and 750 bp, respectively. The best maximum likelihood tree and Bayesian consensus phylogram had similar topologies: *Aegista oculus* from Miyako Island formed a monophyly outside the other three species (Figure 2). *Aegista vermis* and *Aegista caerulea* from Ishigaki Island formed a sister pair with high branch support except for one individual of *Aegista vermis* from Tozato, Ishigaki Island (sampling site 32), which was found in an unresolved position in Bayesian phylogeny. The cluster of *Aegista subchinensis* revealed a basal bifurcation between eastern and western populations. The western and eastern *Aegista subchinensis* formed reciprocal monophyletic clades with highly supported by the bootstrap, aLRT and Bayesian posterior probability. However, the monophyly of the *Aegista subchinensis* clade was only weakly supported. Low statistical branch support for the monophyly of the *Aegista subchinensis* clade probably resulted from conflicting phylogenetic relationships between the COI and the 16S gene tree and unresolved phylogenetic relationships among species of the ITS2 gene tree (Suppl. material 1, Figures S1–6). Maximum likelihood and Bayesian COI gene trees suggested that the eastern *Aegista subchinensis* was the sister clade of *Aegista vermis* and the western *Aegista subchinensis* (Suppl. material 1, Figures S1 and S4). Maximum likelihood 16S gene tree showed sister relationship between the western and the eastern *Aegista subchinensis* with low branch support (Suppl. material 1, Figure S2), but the eastern *Aegista subchinensis* was sister species to *Aegista vermis* with moderate branch support in Bayesian tree (Suppl. material 1, Figure S5). COI gene tree estimated by maximum likelihood and Bayesian inference and the 16S Bayesian gene tree suggested that the western and the eastern *Aegista subchinensis* were not sister clades.

**Figure 3.**
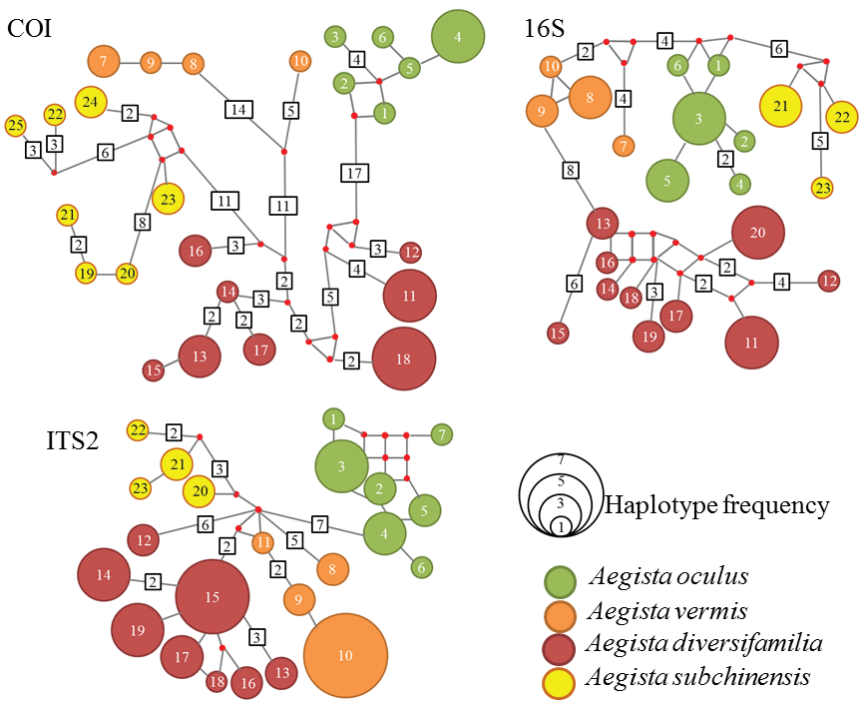
Haplotype networks of mitochondrial COI, 16S and nuclear ITS2 genes. Species are presented by colors. Haplotype frequency is shown by the size of the circular. Name of haplotype is numbered and presented inside the circle. Haplotypes are connected by simple line represent one mutation between haplotypes. Number of mutations between haplotypes are shown in square. Red dots are missing or hypothetical haplotypes.

No haplotypes were shared among species for COI, 16S or ITS2 genes (Figure 3). For COI haplotype network, the eastern *Aegista subchinensis* was nested with the other three species for at least 16 mutations, no clear sister species relationship could be inferred. Haplotype network of 16S gene suggested the western and the eastern *Aegista subchinensis* were distant related. The western *Aegista subchinensis* was nested with *Aegista oculus* at least 9 mutations. The eastern *Aegista subchinensis* was nested with *Aegista vermis* at least 8 mutations. For ITS2 haplotype network, the eastern *Aegista subchinensis* was more closely related to *Aegista vermis* that separated by at least 3 mutations. The eastern and western *Aegista subchinensis* were diverged at least 5 mutations.

Gene trees and haplotype networks suggested the western and the eastern *Aegista subchinensis* were not sister clades. The eastern *Aegista subchinensis* was more closely related to *Aegista vermis* that distributed in Ishigaki Island, Japan. The absence of shared haplotype between the eastern and the western *Aegista subchinensis* might suggest that they were diverged and currently no gene flow between them.

The mean genetic distance between the western and the eastern *Aegista subchinensis* clades was 5.9% for COI, 4.2% for 16S, and 0.8% for ITS2. This divergence corresponded to the divergence between other closely related congeneric species (Table 2). Based on phylogenetic trees, haplotype networks and genetic distance analyses, the western and the eastern *Aegista subchinensis* were diverged and might not be sister clades. More genetic markers are needed to resolve the low statistical support of phylogenetic relationships among species.

**Table 2.** Interspecific divergence and intraspecific polymorphism of *Aegista* spp. from COI, 16S and ITS2 genes.

*COI/16S/ITS2*	*Aegista oculus*	*Aegista vermis*	*Aegista diversifamilia*	*Aegista subchinensis*
*Aegista oculus*	0.007/0.005/0.002			
*Aegista vermis*	0.075/0.036/0.015	0.002/0.008/0.004		
*Aegista diversifamilia*	0.067/0.051/0.014	0.064/0.034/0.007	0.023/0.014/0.004	
*Aegista subchinensis*	0.085/0.037/0.016	0.065/0.038/0.009	0.059/0.042/0.008	0.024/0.012/0.004

### Morphological analyses

**Figure 4.**
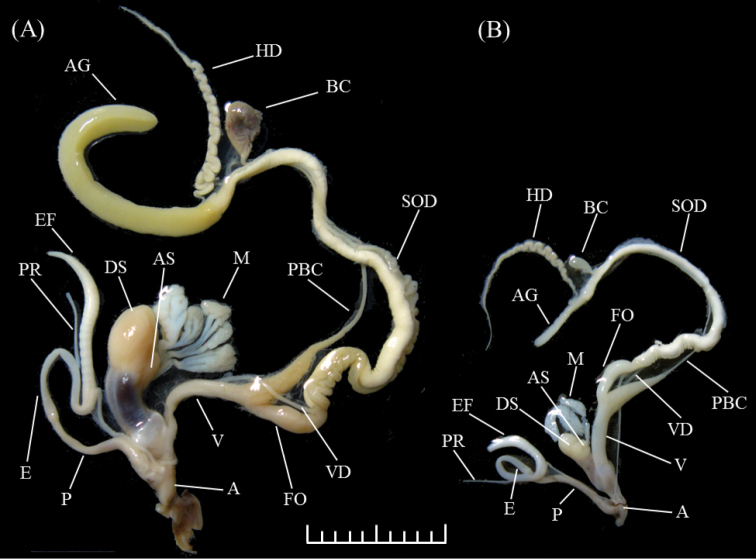
Genital morphology of **A** *Aegista diversifamilia* sp. n. and **B** *Aegista subchinensis*. Scale bar = 1 cm. **A:** atrium; **AG:** albumen gland; **AS:** accessory-sac of auxiliary copulatory organ; **BC:** bursa copulatory; **DS:** dart-sac of auxiliary copulatory organ; **E:** epiphallus; **EF:** epiphallial flagellum; **FO:** free oviduct; **HD:** hermaphroditic duct; **M:** gland of the auxiliary copulatory organ; **P:** penis; **PBC:** pedunculus of bursa copulatory; **PR:** penis retractor; **SOD:** spermoviduct; **V:** vagina; **VD:** vas deferens.

The major differences of genital morphology of the western and the eastern *Aegista subchinensis* were the length of AG, DS, EF, the shape of DS, and the number of lobes of mucus gland in the auxiliary copulatory organ (M) (Figure 4, Table 3). The length of AG of the eastern *Aegista subchinensis* (0.88, scaled by shell width) was three times longer than the western (0.24). The length of DS and EF were nearly two times longer in the eastern *Aegista subchinensis* (DS=0.45, EF=0.57) than the western (DS=0.23, EF=0.29). The shape of DS was more rounded and larger in the eastern *Aegista subchinensis* than the western. The eastern *Aegista subchinensis* had three lobes of M and the western *Aegista subchinensis* had two (Figure 4). The HD/AG ratio was the most different characteristics between the eastern and the western *Aegista subchinensis* that showed HD was three times longer than AG in the western *Aegista subchinensis* but HD was shorter than AG in the eastern (Table 3). The eastern *Aegista subchinensis* showed larger values than the western in AG/SOD, EF/E and E/P ratio.

**Table 3.** Measurements for genital morphology of *Aegista subchinensis* and *Aegista diversifamilia* **sp. n.** The measurements are scaled by shell width, *Aegista subchinensis* (1.9 cm, collected from Zhishanyan, Taipei City) and *Aegista diversifamilia* **sp. n.** (2.3 cm, collected from Heren 1, Xiulin Township, Hualien County)

Measurement	HD	AG	SOD	FO	V	DS	P	E	EF
*Aegista subchinensis*	0.77	0.24	1.05	0.20	0.20	0.23	0.34	0.32	0.29
*Aegista diversifamilia*	0.73	0.88	1.38	0.22	0.33	0.45	0.33	0.45	0.57
**Ratio**	HD/AG	AG/SOD	HD/SOD	FO/V	EF/E	E/P	P/V		
*Aegista subchinensis*	3.19	0.23	0.73	1.00	0.89	0.94	1.75		
*Aegista diversifamilia*	0.84	0.63	0.53	0.66	1.25	1.37	1.01		

AG: albumen gland; DS: dart-sac of auxiliary copulatory organ; E: epiphallus; EF: epiphallial flagellum; FO: free oviduct; HD: hermaphroditic duct; P: penis; SOD: spermoviduct; V: vagina.

**Figure 5.**
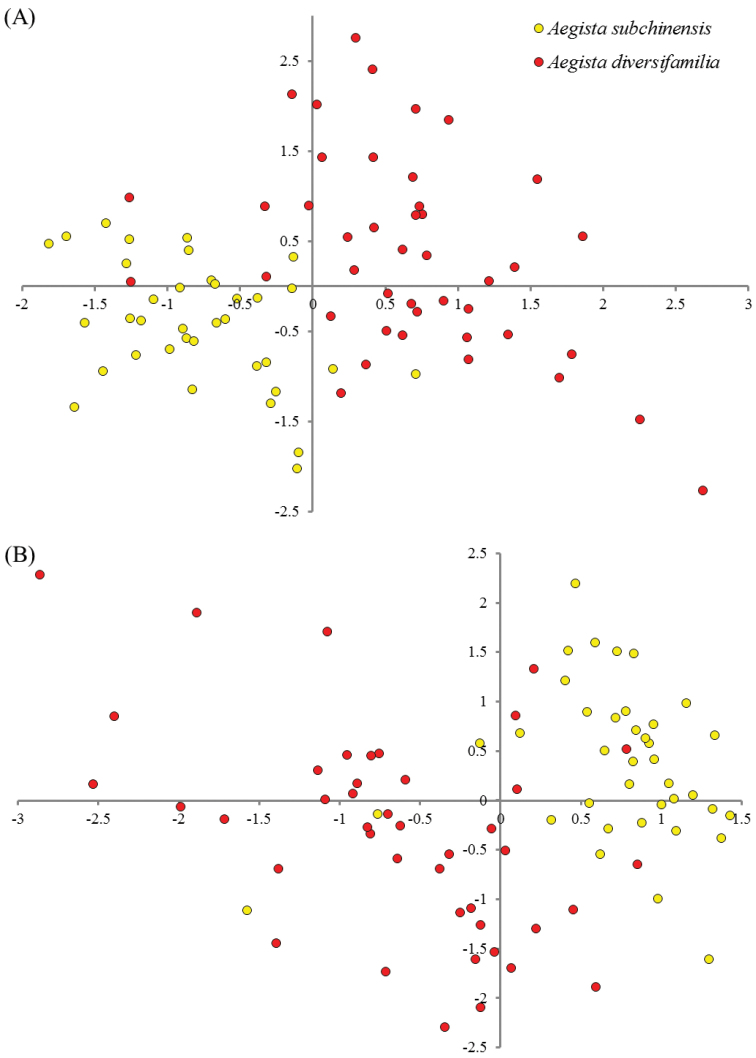
Principle component analysis (PCA) of *Aegista subchinensis* and *Aegista diversifamilia* sp. n. **A** PCA of measurements and ratios **B** relative warps PCA of shell shape coordinates.

**Figure 6.**
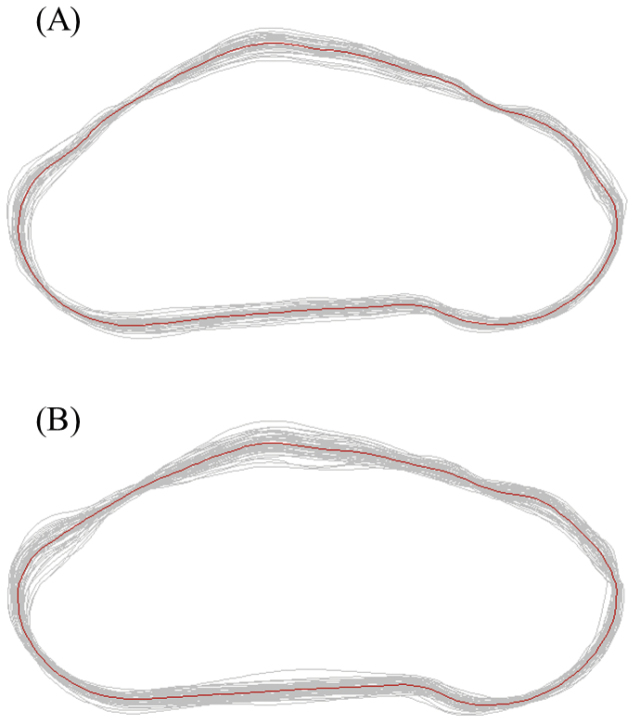
Mean shell shape of **A** *Aegista subchinensis* and **B** *Aegista diversifamilia* sp. n.

**Figure 7.**
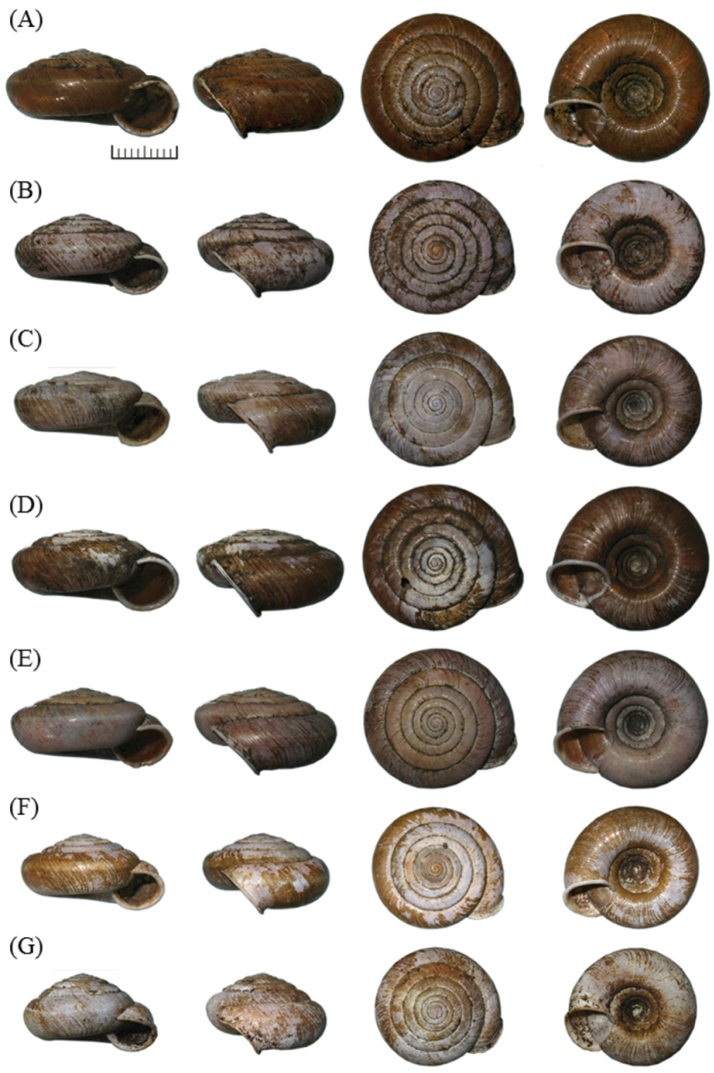
Shell images of *Aegista diversifamilia* sp. n. and *Aegista subchinensis*. *Aegista diversifamilia* sp. n.: **A** holotype, NMNS-7276-001 **B–E** paratype, NHMUK20140070, the same locality of holotype. *Aegista subchinensis*: **F** collected from Zhishanyan, Taipei City **G** collected from Linmei Shipan Trail, Jiaoxi Township, I-Lan County. Scale bar = 1 cm.

**Figure 8.**
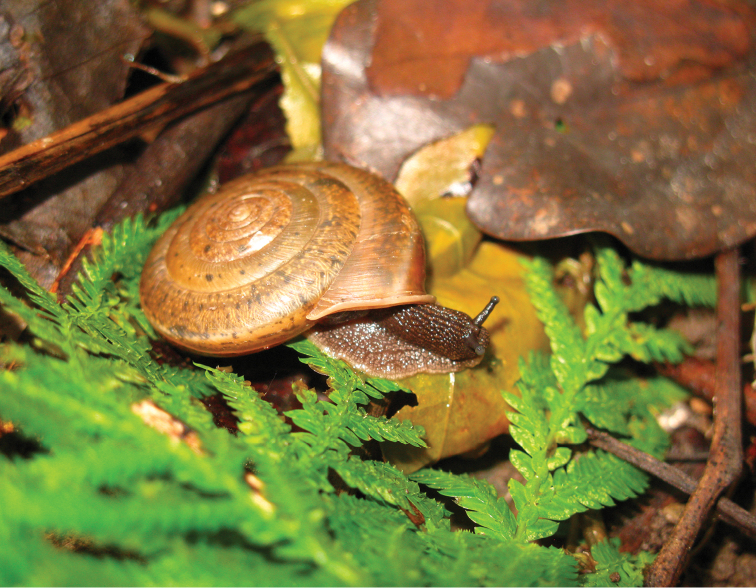
Living snail *Aegista diversifamilia* sp. n. from Heren, Xiulin Township, Hualien County, Taiwan (sampling site 19 in Table 1).

The eastern and western populations of *Aegista subchinensis* differed significantly from each other in all studied shell parameters (*p* < 0.001) except the number of whorls and the height of the secondary body whorl (Table 4, Suppl. material 1, Table S1). The eastern *Aegista subchinensis* had a similar number of whorls (*p* > 0.05) but shells were significantly larger than those of the western *Aegista subchinensis* and had a wider apex angle. The eastern and western populations also differed significantly in all morphometric ratios except UW/SW, AW/UW, 2W/3W, 3W/4W and 5W/6W. MANOVA suggested that the morphological difference between the western and the eastern *Aegista subchinensis* was statistical significant (Bonferroni-corrected *p*-value=1.12E-10). Results of the PCA of morphological characteristics suggested that principle component axis 1 (PC1) explained 46.32% of the total variation and had the highest loading scores for aperture height (0.284). PC2 explained 20.03% of the total variation and had the highest loading scores for height of the secondary body whorl (0.476). The PCA scores plot of PC1 and PC2 showed that morphology between the eastern and the western *Aegista subchinensis* were well distinguished with very limited overlap (Figure 5A). Discriminant analysis showed that specimens could be correctly classified (100%) into eastern and western clades (93.67% using Jackknifed analysis). Relative warps PCA of shell outline coordinates suggested that PC1 and PC2 represented 94.43% and 3.07% of the total variation, respectively. The scores plot of relative warps PC1 and PC2 also showed prominent morphological differences between the eastern and the western clade (Figure 5B). The mean shape of the western and the eastern *Aegista subchinensis* were presented in Figure 6. The eastern *Aegista subchinensis* (Figure 6B) was more flat than the western *Aegista subchinensis* (Figure 6A). The type locality of *Aegista subchinensis* was Tamsui in northern Taiwan (Figure 2). The illustration of *Aegista subchinensis* presented in fig 7 figure 8 of the original description (Möllendorff 1884) showed higher conic shape that was similar to our analysed western populations of *Aegista subchinensis*. Despite we did not have sample from Tamsui, the type locality of *Aegista subchinensis*, we sampled much wider geographical region around Tamsui that included more variation of molecular and morphological characteristics.

**Table 4.** Measurements (in cm) of *Aegista subchinensis* and *Aegista diversifamilia* sp. n. Mean, standard error, statistical method and the *p*-value were provided.

	***Aegista subchinensis*** (N=36)	***Aegista diversifamilia*** (N=43)	
	**Mean±SE**	**Mean±SE**	**Statistical difference**
whorls	7.21±0.04	7.23±0.05	M
shell width (SW)	1.97±0.02	2.46±0.04	W, *p*=9.80E-16
shell height (SH)	1.04±0.01	1.21±0.02	M, *p*=2.09E-6
aperture width (AW)	0.75±0.01	0.97±0.02	W, *p*=1.23E-15
aperture height (AH)	0.53±0.01	0.74±0.02	W, *p*=3.47E-15
umbilicus width (UW)	0.72±0.01	0.95±0.02	M, *p*=3.33E-13
body whorl height (BH)	0.57±0.01	0.68±0.01	W, *p*=3.83E-11
secondary body whorl height (SBH)	0.10±0.00	0.10±0.00	M
Angle of apex (AA)	150.8±0.65	154.7±0.67	M, *p*=2.23E-4
First whorl width (FW)	0.14±0.00	0.16±0.00	M, *p*=5.77E-5
2^nd^ whorl width (2W)	0.06±0.00	0.07±0.00	M, *p*=1.21E-5
3^rd^ whorl width (3W)	0.08±0.00	0.10±0.00	M, *p*=9.74E-11
4^th^ whorl width (4W)	0.12±0.00	0.14±0.00	M, *p*=8.23E-8
5^th^ whorl width (5W)	0.16±0.00	0.19±0.00	M, *p*=6.37E-12
6^th^ whorl width (6W)	0.20±0.00	0.25±0.00	M, *p*=4.03E-11
SH/SW	0.53±0.01	0.49±0.00	W, *p*=7.71E-9
AW/SW	0.38±0.00	0.39±0.00	M, *p*=2.37E-3
UW/SW	0.37±0.00	0.39±0.01	M
AH/SH	0.51±0.01	0.62±0.01	W, *p*=9.84E-12
AH/AW	0.70±0.01	0.76±0.01	W, *p*=9.18E-6
SH/UW	1.45±0.02	1.28±0.03	M, *p*=5.42E-6
AW/UW	1.05±0.01	1.03±0.02	M
AH/UW	0.73±0.01	0.79±0.02	M, *p*=7.21E-4
BH/SW	0.29±0.00	0.28±0.00	M, *p*=4.70E-6
BH/SH	0.55±0.00	0.57±0.01	W, *p*=4.40E-3
BH/UW	0.79±0.01	0.73±0.02	M, *p*=2.43E-3
SBH/BH	0.68±0.01	0.78±0.01	W, *p*=7.14E-9
SBH/SW	0.05±0.00	0.04±0.00	W, *p*=6.84E-7
SBH/SH	0.10±0.00	0.08±0.00	W, *p*=2.35E-5
SBH/UW	0.15±0.01	0.11±0.00	W, *p*=2.76E-8
FW/SW	0.07±0.00	0.06±0.00	M, *p*=4.80E-6
2W/3W	0.75±0.03	0.68±0.01	M
3W/4W	0.68±0.02	0.72±0.01	M
4W/5W	0.76±0.01	0.70±0.01	M, *p*=6.09E-4
5W/6W	0.79±0.01	0.77±0.01	W

M: Mann-Whitney *U* test; W: Welch’s *t* test.

Based on the observed amounts of morphological and genetic differentiation, we conclude that the eastern and western populations assigned to *Aegista subchinensis* have diverged into separate species. Phylogeny reconstructed from concatenated sequences supports monophyly of both clades corresponding to their allopatric distributional pattern that separated by the Lanyang River. The Lanyang River was a biogeographic barrier for a high elevation mammal, Formosan wood mouse *Apodemos semotus* (Hsu et al. 2001). Some researchers identified the Xueshan Mountain Range, located in the northern area of Lanyang River, as a biogeographic barrier for lowland animals (Lin et al. 2012, Shih et al. 2011, Yu et al. 2014). To our knowledge, this is the first study revealed that the Lanyang River as a barrier for lowland terrestrial animals. We suggested that the eastern *Aegista subchinensis* might be diverged from the western *Aegista subchinensis* by vicariance event.

## Systematics

### Superfamily Helicoidea Rafinesque, 1815 Family Bradybaenidae Pilsbry, 1939

#### Genus Aegista

Taxon classificationAnimaliaStylommatophoraBradybaenidae

Albers, 1850

##### Type species.

*Helix chinensis* Philippi, 1845, original designation.

#### Aegista diversifamilia sp. n.

Taxon classificationAnimaliaStylommatophoraBradybaenidae

http://zoobank.org/B36A2814-2702-40B0-844B-AC4B12A8BD25

Figures 7 , 8 , Table 4 , Suppl. material 1, Table S1

Aegista subchinensis Hsieh, 2003: 200, figs; Lee and Chen 2003: 234, figs above text, figs 1–2; Lee and Wu 2004: 13–14, figures 2A, 3D; Hsieh et al. 2006: 250, figs; Wu and Jian 2006: fig 33; Hsieh et al. 2013: 335, figs.
Aegista (Aegista) subchinensis Hemmen and Niederhöfer, 2007: figs 67, figs 80; Wen and Hwang 2014: fig 1.

##### Type material.

**Holotype** NMNS-7276-001 (adult dry shell, Figure 7A). **Paratypes** NMNH-7276-002 (1 juvenile in EtOH) and NHMUK 20140070 (4 adult dry shells, Figure 7B–E) from the same locality of holotype. NMNH-7276-003 (1 adult dry shell) and NMNH-7276-004 (1 adult dry shell) from the northern entrance of Chongde Tunnel, Xiulin Township, Hualian County, 24°11'31.08"N, 121°39'41.01"E, elevation 62 m. NMNS-7276-005 (6 adult dry shells) and NHMUK 20140071 (2 adult dry shells) from Jinwen Tunnel, Xiulin Township, 24°12'28.7"N, 121°40'23.5"E, elevation 128m.

##### Type locality.

Taiwan, Hualian County, Xiulin Township, Forest around the Chongde Tunnel, 24°11'22.0"N, 121°39'36.8"E, elevation 56 m.

##### Other material examined.

Anpingkeng, Dongshan Township, I-Lan County, 24°36'52.5"N, 121°46'38.1"E (3 adult dry shells); Wushibi, Su’ao Township, 24°29'13.5"N, 121°50'02.9"E (1 juvenile in EtOH); Chaoyang Trail, Nan’ao Township, 24°27'35.9"N, 121°48'53.9"E (2 adult dry shells); Heren 1, Xiulin Township, Hualien County, 24°14'49.1"N, 121°43'06.4"E (1 adult dry shells); Heren 2, 24°14'54.8"N, 121°42'51.4"E (7 adult dry shells); Heren Trail, 24°13'58.5"N, 121°42'27.73"E (1 adult and 4 juvenile in EtOH); Southern Chongde Tunnel, 24°11'22.0"N, 121°39'36.8"E (2 juvenile in EtOH); Sanjianwu, 24°10'55.3"N, 121°37'34.3"E (6 adult dry shells); Taroko Service Center, 24°09'31.9"N, 121°37'20.7"E (6 adult dry shells); Badagang, 24°10'36.8"N, 121°33'43.6"E (1 adult and 4 juvenile in EtOH, 6 adult dry shells) (materials mentioned above were deposited in NMNS, NMNH-7276); Hoping Forest Road, (1 adult in EtOH, NMNS-004875-00015 and 1 adult dry shell, NMNS-004962-00038); Sanzhan Northern Stream, (1 adult dry shell, NMNS-003348-00023).

##### Description.

*Shell Morphology.* Shell depressed globosed, dextral, medium sized, shell width range 1.98–3.24 cm, shell height range 0.97–1.68 cm, shell height/shell width ratio range 0.43–0.55. Shell thin but solid, glossy with chestnut brown or yellowish-brown, usually with narrow and light brown spiral band on periphery. Shell surface with distinct oblique and curved growth lines. Apex obtuse, angle range 148.56°–165.02°. Spire depressed conic, slightly convex, suture depressed. Whorl range 6.6–8.2 in number, earlier whorl narrow then slowly increases regularly, and last whorl shouldered. Body whorl height range 0.53–0.88 cm. Aperture little descending, ovate or nearly circular, width range 0.78–1.32 cm, height range 0.48–1.05 cm. Peristome white, expanded and reflected. Umbilicus widely open, width range 0.77–1.59 cm. Mean and standard errors of each characteristics were provided in Table 4. Morphological measurements of all specimens were presented in Suppl. material 1, Table S1.

*Genital morphology.* Atrium thick and short. Penis slender and long. Epiphallus slender, longer than penis. Penis retractor muscle thin and long, attached to one-third part of epiphallus. Epiphallial flagellum thick and long, logner than epiphallus, wider than penis and epiphallus. Dart-sac of auxiliary copulatory organ thick and large, inserted into the base of vagina, with one small accessory-sac of auxiliary copulatory organ. Three mucus glands of the auxiliary copulatory organ. Vagina slender at the base of dart-sac, gradual wider and thick toward free oviduct, inflated at the connected region of free oviduct, about equal length of penis. Free oviduct thick, short,inflated. Pedunculus of bursa copulatory thin and long. Sac of bursa copulatory large and oval. Vas deferens thin and long, wider than penis retractor muscle. Spermoviduct long, about four times longer than penis and oviduct. Hermaphroditic duct slender and long, about half length of spermoviduct. Albumen gland thick and long, longer than hermaphroditic duct.

##### Etymology.

Named after the recent efforts supporting equal marriage rights in Taiwan and around the world. Derived from “diversus” (Latin for different) and “familia” (Latin for family), adjective of feminine gender.

##### Distribution.

Endemic to Taiwan and is currently known from I-Lan and Hualian Counties. *Aegista diversifamilia* sp. n. is absent from Gueishan Island based on our field investigation (Huang et al. 2013). The northernmost distribution is limted by the Lanyang River. We suggest that the Lanyang River is the putative biogeographic boundary between *Aegista diversifamilia* sp. n. and *Aegista subchinensis*.

##### Ecology.

Live snails are generally found on the ground or under leaf litter in shady, moist environments in lowland hardwood forests (Figure 8). Eggs white and round, approximately 3 mm in diameter with 20–30 eggs in each spawn (personal observation of reared snail in laboratory).

##### Remarks.

*Aegista diversifamilia* sp. n. can be distinguished from *Aegista subchinensis* by its overall larger shell width (1.98–3.24 cm), whorl width and aperture, more depressed shell, and wider umbilicus (0.77–1.59 cm) and larger apex angle (148.56°–165.02°) (see Suppl. material 1, Table S1). For the genital morphology, *Aegista diversifamilia* sp. n. was distinguished from *Aegista subchinensis* by thicker and about three times logner albumen gland, larger and about two times longer dart-sac of auxiliary copulatory organ and epiphallial flagellum. The length of hermaphroditic duct/ albumen gland ratio was three times larger in *Aegista diversifamilia* sp. n. than in *Aegista subchinensis*.

The morphological divergence between the eastern and the western *Aegista subchinensis* was firstly noticed by Lee and Chen (2003), who found that the shells from the western population were roughly one third smaller than those from the eastern population. When newly describing *Aegista caperata*, Lee and Wu (2004) suggested the presence of cryptic species within *Aegista subchinensis* from different sides of the CMR. Wen and Hwang (2014) compared reproductive system between subgenus *Aegista* and *Plectotropis*. Aegista (Aegista) subchinensis and Aegista (Plectotropis) mackensii were dissected as representative species. Wen and Hwang (2014) mentioned there were two lobes of mucus glands of *Aegista subchinensis*. According to the shell masurements, sampling locality (Xiulin Township, Hualien County) and the illustration of genital morphology figure 1 of *Aegista subchinensis* was actually the nominal new species presented here, *Aegista diversifamilia* sp. n. It might suggested that the number of lobes of mucus glands is a variable characteristic in *Aegista diversifamilia* sp. n.

## Supplementary Material

XML Treatment for Aegista

XML Treatment for Aegista diversifamilia

## Supplementary materials

Supplementary material 1 Gene trees of maximum likelihood and Bayesian inference and the morphological measurements of *Aegista diversifamilia* sp. n. and *Aegista subchinensis*. Data type: phylogenetic tree/measurement Explanation note: Figure S1. Maximum likelihood phylogeny of mitochondrial COI gene. Branch support confidences are shown in bootstrap and approximate likelihood-ratio test. Figure S2. Maximum likelihood phylogeny of mitochondrial 16S gene. Branch support confidences are shown in bootstrap and approximate likelihood-ratio test. Figure S3. Maximum likelihood phylogeny of nuclear ITS2 gene. Branch support confidences are shown in bootstrap and approximate likelihood-ratio test. Figure S4. Bayesian phylogeny of mitochondrial COI gene. Figure S5. Bayesian phylogeny of mitochondrial 16S gene. Figure S6. Bayesian phylogeny of nuclear ITS2 gene. Table S1. Morphological measurements of *Aegista diversifamilia* sp. n. and *Aegista subchinensis*. This dataset is made available under the Open Database License (http://opendatacommons.org/licenses/odbl/1.0/). The Open Database License (ODbL) is a license agreement intended to allow users to freely share, modify, and use this Dataset while maintaining this same freedom for others, provided that the original source and author(s) are credited.Chih-Wei Huang, Yen-Chen Lee, Si-Min Lin, Wen-Lung Wu
