# Supplementary material for: Taxonomic revision of Aegista subchinensis (Möllendorff, 1884) (Stylommatophora, Bradybaenidae) and a description of a new species of Aegista from eastern Taiwan based on multilocus phylogeny and comparative morphology
Source: Zookeys. 2014 Oct 13;(445):31–55. doi: 10.3897/zookeys.445.7778 (PMC4205739; doi:10.3897/zookeys.445.7778)
Supplement: Supplementary material 1 — Gene trees of maximum likelihood and Bayesian inference and the morphological measurements of Aegista diversifamilia sp. n. and Aegista subchinensis. [file zookeys-445-031-s001.doc]

Supplementary File

Legend

Figure S1. Maximum likelihood phylogeny of mitochondrial COI gene. Branch support confidences are shown in bootstrap and approximate likelihood-ratio test.

Figure S2. Maximum likelihood phylogeny of mitochondrial 16S gene. Branch support confidences are shown in bootstrap and approximate likelihood-ratio test.

Figure S3. Maximum likelihood phylogeny of nuclear ITS2 gene. Branch support confidences are shown in bootstrap and approximate likelihood-ratio test.

Figure S4. Bayesian phylogeny of mitochondrial COI gene.

Figure S5. Bayesian phylogeny of mitochondrial 16S gene.

Figure S6. Bayesian phylogeny of nuclear ITS2 gene.

Table S1. Morphological measurements of *Aegista diversifamilia* **sp. n.** and *A. subchinensis*


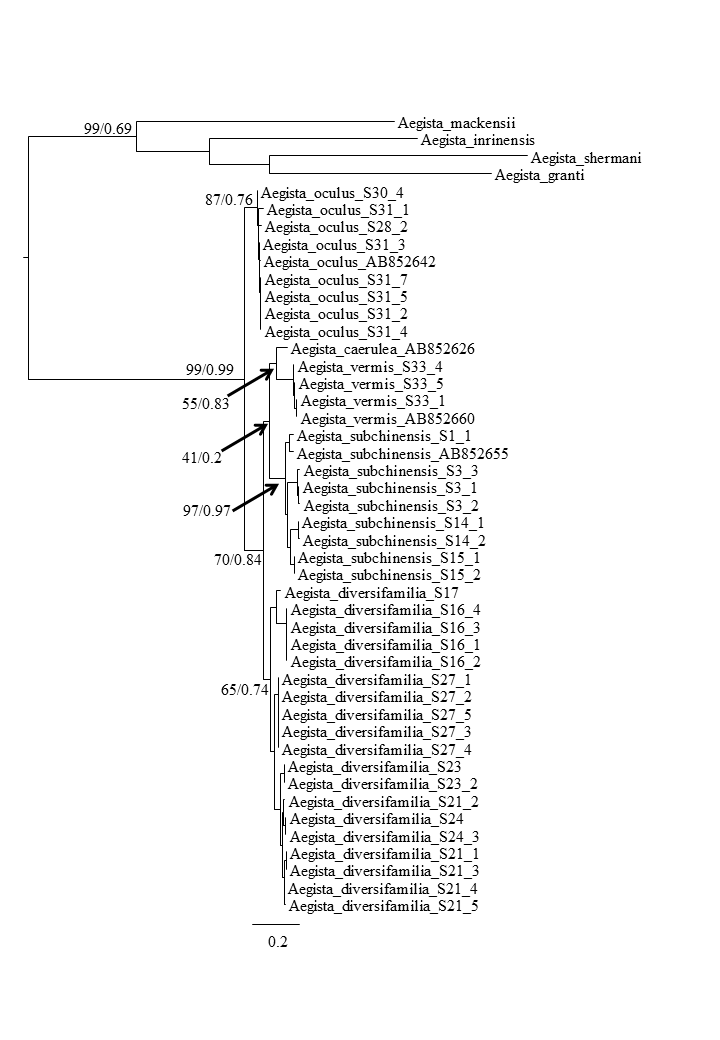


Figure S1.


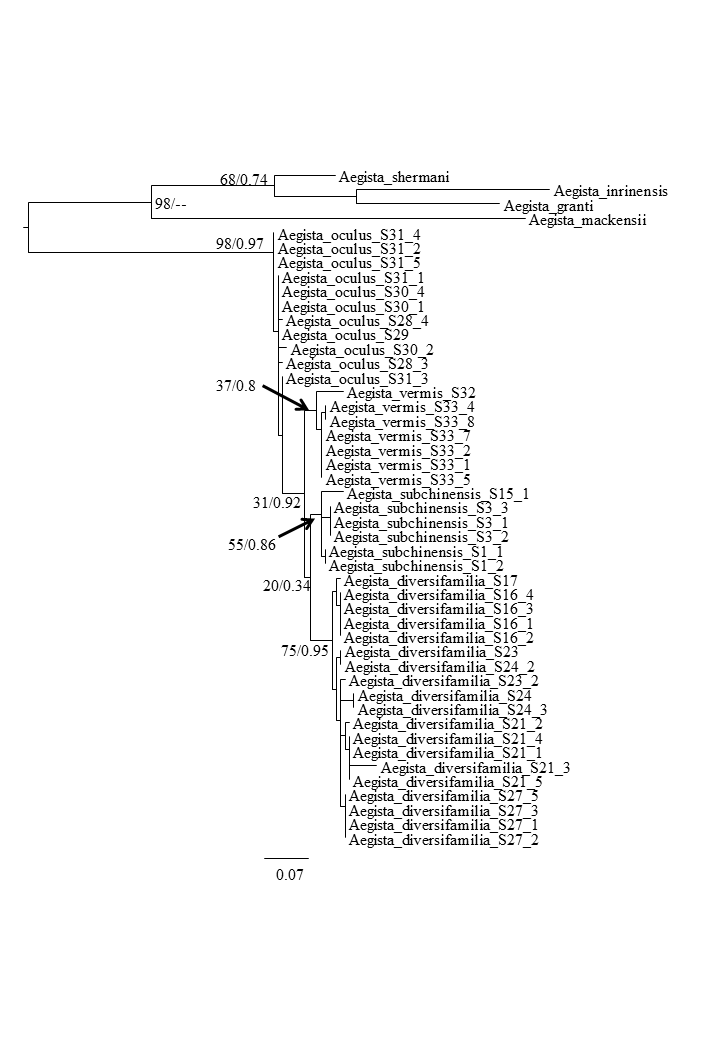


Figure S2.


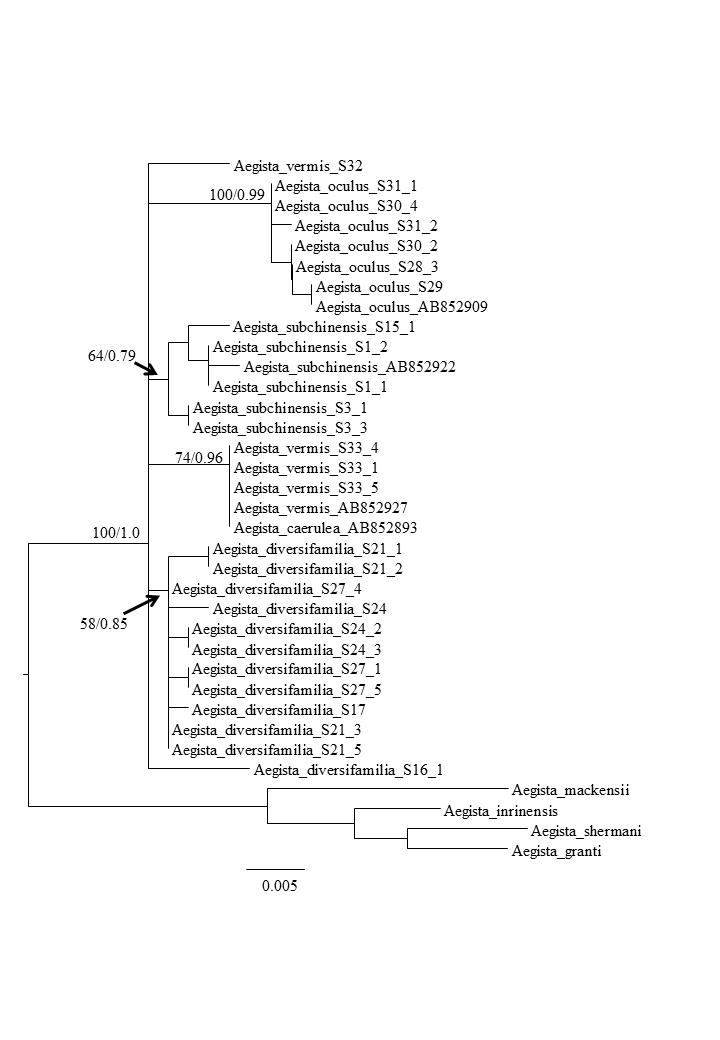


Figure S3.


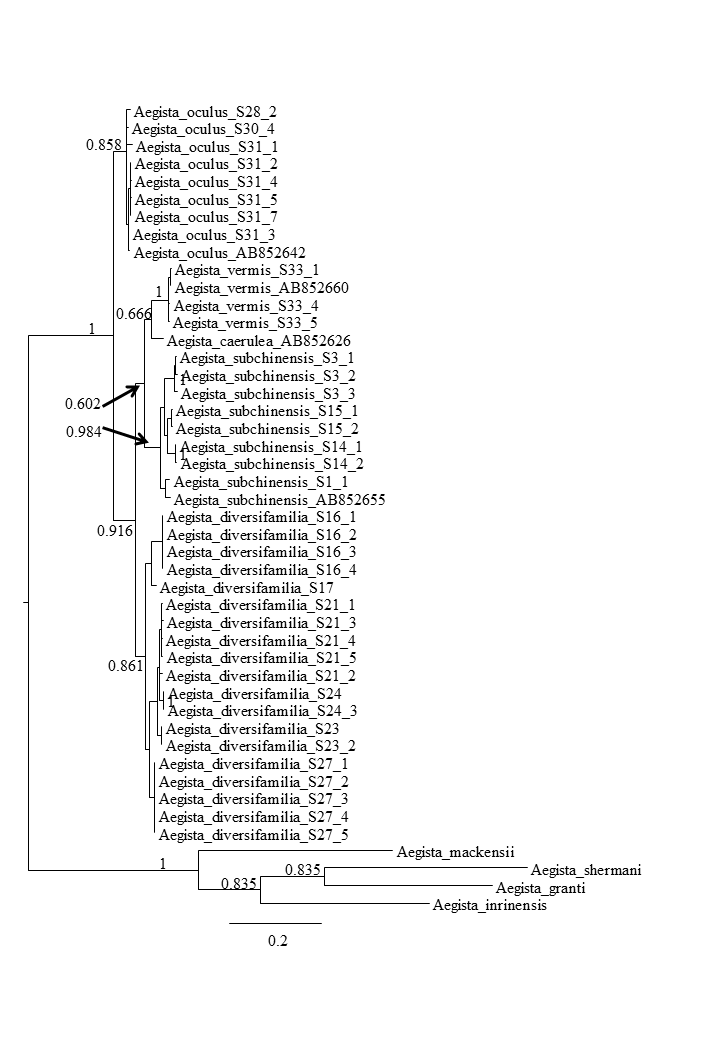


Figure S4.


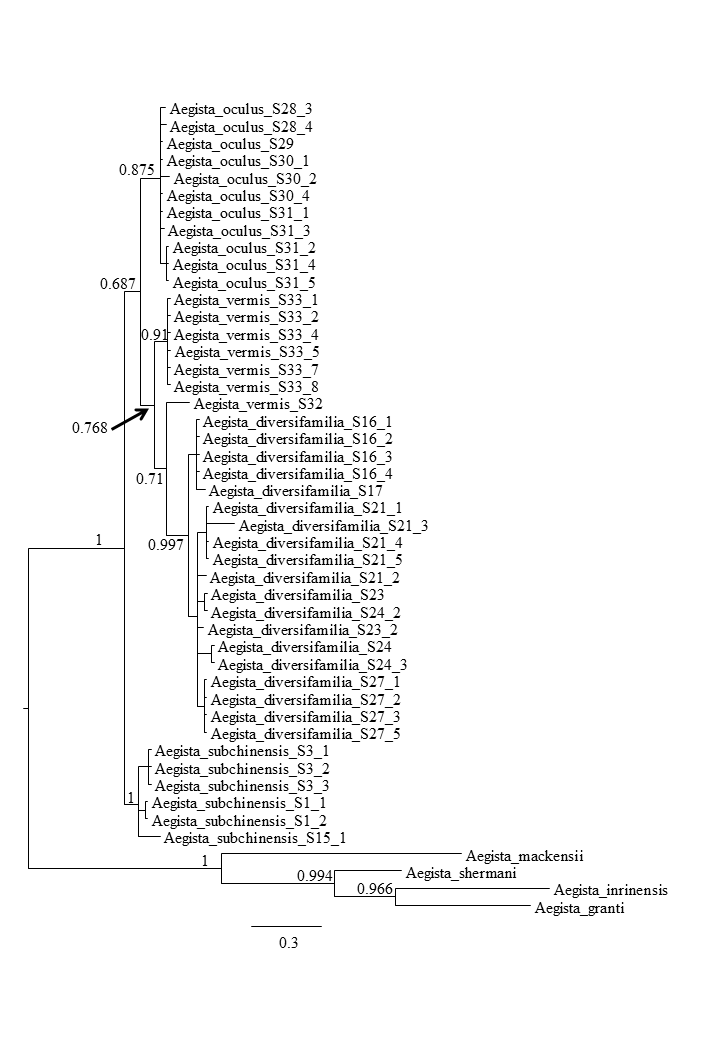


Figure S5.


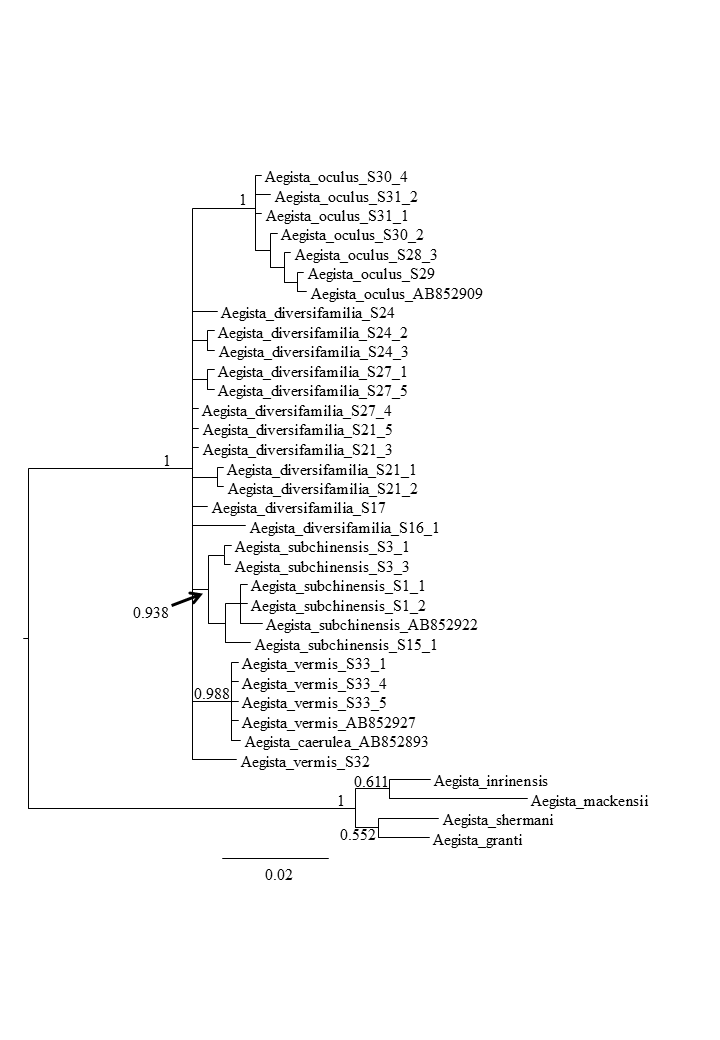


Figure S6.

Table S1. Morphological measurements of *Aegista diversifamilia* sp. n. and *A. subchinensis*

| TYPE | registration number | species | field collection number | sampling site | individual | specimen | whorls | SW | SH | AW | AH | UW | BH | SBH | AA | FW | 2W | 3W | 4W | 5W | 6W |
| --- | --- | --- | --- | --- | --- | --- | --- | --- | --- | --- | --- | --- | --- | --- | --- | --- | --- | --- | --- | --- | --- |
|  |  | *Aegista diversifamilia* | YL20110419-01 | S16 | 1 | dead shell | 7.2 | 2.51 | 1.26 | 0.97 | 0.81 | 0.94 | 0.73 | 0.08 | 149.45 | 0.15 | 0.06 | 0.10 | 0.12 | 0.18 | 0.23 |
|  |  | *Aegista diversifamilia* | YL20110419-01 | S16 | 2 | dead shell | 7.5 | 2.53 | 1.23 | 0.91 | 0.74 | 1.01 | 0.73 | 0.11 | 151.78 | 0.17 | 0.06 | 0.09 | 0.15 | 0.18 | 0.27 |
|  |  | *Aegista diversifamilia* | YL20110419-01 | S16 | 3 | dead shell | 7.6 | 2.56 | 1.31 | 1.01 | 0.77 | 1.00 | 0.74 | 0.13 | 150.24 | 0.15 | 0.08 | 0.10 | 0.14 | 0.18 | 0.23 |
|  |  | *Aegista diversifamilia* | YL20110419-01 | S16 | 2 | EtOH | 7.2 | 2.46 | 1.21 | 0.94 | 0.70 | 1.31 | 0.72 | 0.08 | 156.33 | 0.16 | 0.07 | 0.09 | 0.14 | 0.19 | 0.25 |
|  |  | *Aegista diversifamilia* | YL20110419-01 | S16 | 3 | EtOH | 7.25 | 2.49 | 1.24 | 1.00 | 0.74 | 0.87 | 0.72 | 0.09 | 152.67 | 0.17 | 0.07 | 0.10 | 0.13 | 0.19 | 0.26 |
|  |  | *Aegista diversifamilia* | YL20100815-03 | S18 | 1 | dead shell | 7.75 | 2.86 | 1.58 | 1.20 | 0.95 | 0.98 | 0.83 | 0.14 | 152.46 | 0.14 | 0.06 | 0.10 | 0.15 | 0.20 | 0.28 |
|  |  | *Aegista diversifamilia* | YL20100815-03 | S18 | 2 | dead shell | 7.3 | 2.66 | 1.29 | 1.04 | 0.83 | 0.92 | 0.73 | 0.12 | 157.71 | 0.16 | 0.06 | 0.10 | 0.14 | 0.21 | 0.26 |
|  |  | *Aegista diversifamilia* | HL20100818-03 | S19 | 1 | dead shell | 7.2 | 2.53 | 1.18 | 1.03 | 0.75 | 0.84 | 0.71 | 0.06 | 154.70 | 0.15 | 0.07 | 0.10 | 0.13 | 0.20 | 0.27 |
|  |  | *Aegista diversifamilia* | HL20110418-05 | S20 | 1 | dead shell | 7 | 2.59 | 1.28 | 1.05 | 0.79 | 0.88 | 0.74 | 0.10 | 149.94 | 0.20 | 0.07 | 0.11 | 0.15 | 0.21 | 0.26 |
|  |  | *Aegista diversifamilia* | HL20110418-05 | S20 | 2 | dead shell | 7 | 2.57 | 1.28 | 1.06 | 0.83 | 0.93 | 0.71 | 0.08 | 153.94 | 0.17 | 0.07 | 0.11 | 0.15 | 0.19 | 0.29 |
|  |  | *Aegista diversifamilia* | HL20110418-05 | S20 | 3 | dead shell | 7.65 | 2.66 | 1.41 | 1.05 | 0.81 | 0.95 | 0.71 | 0.15 | 148.56 | 0.16 | 0.06 | 0.11 | 0.14 | 0.19 | 0.24 |
|  |  | *Aegista diversifamilia* | HL20110418-05 | S20 | 4 | dead shell | 8 | 3.10 | 1.54 | 1.20 | 0.93 | 1.12 | 0.84 | 0.13 | 155.72 | 0.13 | 0.07 | 0.09 | 0.13 | 0.18 | 0.26 |
|  |  | *Aegista diversifamilia* | HL20110418-05 | S20 | 5 | dead shell | 8.2 | 3.24 | 1.68 | 1.32 | 0.87 | 1.16 | 0.88 | 0.16 | 149.15 | 0.16 | 0.06 | 0.10 | 0.13 | 0.20 | 0.23 |
|  |  | *Aegista diversifamilia* | HL20110418-05 | S20 | 6 | dead shell | 7.75 | 2.94 | 1.40 | 1.06 | 1.05 | 1.18 | 0.80 | 0.10 | 158.50 | 0.16 | 0.07 | 0.10 | 0.13 | 0.21 | 0.26 |
|  |  | *Aegista diversifamilia* | HL20110418-05 | S20 | 7 | dead shell | 7.65 | 2.86 | 1.37 | 1.17 | 0.98 | 1.06 | 0.79 | 0.12 | 155.18 | 0.14 | 0.06 | 0.11 | 0.14 | 0.19 | 0.27 |
|  |  | *Aegista diversifamilia* | HL20110603 | S21 | 1 | EtOH | 7.14 | 2.49 | 1.28 | 1.03 | 0.80 | 0.90 | 0.71 | 0.10 | 153.38 | 0.15 | 0.07 | 0.10 | 0.15 | 0.20 | 0.28 |
| Paratype | NMNS-7276-005 | *Aegista diversifamilia* | HL20100818-02 | S22 | 1 | dead shell | 6.8 | 2.27 | 1.12 | 0.93 | 0.83 | 0.86 | 0.66 | 0.06 | 156.33 | 0.15 | 0.06 | 0.09 | 0.14 | 0.20 | 0.26 |
| Paratype | NMNS-7276-005 | *Aegista diversifamilia* | HL20100818-02 | S22 | 2 | dead shell | 6.75 | 2.34 | 1.08 | 0.95 | 0.70 | 0.80 | 0.67 | 0.08 | 163.75 | 0.17 | 0.07 | 0.10 | 0.16 | 0.23 | 0.28 |
| Paratype | NMNS-7276-005 | *Aegista diversifamilia* | HL20100818-02 | S22 | 3 | dead shell | 7 | 2.33 | 1.05 | 0.94 | 0.80 | 0.87 | 0.65 | 0.04 | 159.51 | 0.14 | 0.06 | 0.09 | 0.13 | 0.19 | 0.27 |
| Paratype | NMNS-7276-005 | *Aegista diversifamilia* | HL20100818-02 | S22 | 4 | dead shell | 7 | 2.36 | 1.11 | 0.98 | 0.69 | 0.77 | 0.69 | 0.07 | 157.89 | 0.16 | 0.06 | 0.09 | 0.14 | 0.21 | 0.26 |
| Paratype | NMNS-7276-005 | *Aegista diversifamilia* | HL20100818-02 | S22 | 5 | dead shell | 6.6 | 2.40 | 1.03 | 0.97 | 0.73 | 0.84 | 0.69 | 0.05 | 165.02 | 0.16 | 0.08 | 0.10 | 0.16 | 0.23 | 0.34 |
| Paratype | NMNS-7276-005 | *Aegista diversifamilia* | HL20100818-02 | S22 | 6 | dead shell | 6.9 | 2.33 | 1.12 | 0.97 | 0.66 | 0.79 | 0.65 | 0.08 | 154.13 | 0.17 | 0.06 | 0.10 | 0.15 | 0.19 | 0.27 |
| Paratype | NHMUK-20140071 | *Aegista diversifamilia* | HL20100818-02 | S22 | 7 | dead shell | 7.1 | 2.52 | 1.22 | 1.02 | 0.81 | 0.92 | 0.74 | 0.10 | 153.99 | 0.16 | 0.06 | 0.10 | 0.14 | 0.21 | 0.26 |
| Paratype | NHMUK-20140071 | *Aegista diversifamilia* | HL20100818-02 | S22 | 8 | dead shell | 6.9 | 2.25 | 1.03 | 0.90 | 0.69 | 0.82 | 0.62 | 0.07 | 157.02 | 0.15 | 0.07 | 0.10 | 0.13 | 0.19 | 0.26 |
| Paratype | NMNS-7276-004 | *Aegista diversifamilia* | 2009Liwu River | S23 | 1 | EtOH | 7 | 2.82 | 1.28 | 1.17 | 0.92 | 1.02 | 0.78 | 0.08 | 161.73 | 0.17 | 0.08 | 0.11 | 0.16 | 0.24 | 0.30 |
| Paratype | NMNS-7276-003 | *Aegista diversifamilia* | HL20101129-01 | S23 | 1 | EtOH | 7.25 | 2.40 | 1.14 | 0.92 | 0.74 | 0.99 | 0.65 | 0.10 | 155.78 | 0.14 | 0.07 | 0.10 | 0.13 | 0.20 | 0.27 |
| Paratype | NHMUK-20140070 | *Aegista diversifamilia* | HL20100818-01 | S24 | 1 | dead shell | 7.4 | 2.36 | 1.26 | 0.90 | 0.62 | 0.83 | 0.66 | 0.12 | 149.28 | 0.15 | 0.06 | 0.10 | 0.13 | 0.19 | 0.22 |
| Paratype | NHMUK-20140070 | *Aegista diversifamilia* | HL20100818-01 | S24 | 2 | dead shell | 7.1 | 2.39 | 1.18 | 0.83 | 0.68 | 0.96 | 0.67 | 0.08 | 157.19 | 0.16 | 0.06 | 0.11 | 0.13 | 0.21 | 0.27 |
| Paratype | NHMUK-20140070 | *Aegista diversifamilia* | HL20100818-01 | S24 | 3 | dead shell | 7.2 | 2.57 | 1.26 | 1.02 | 0.84 | 0.95 | 0.72 | 0.11 | 163.09 | 0.17 | 0.07 | 0.11 | 0.15 | 0.23 | 0.26 |
| Paratype | NHMUK-20140070 | *Aegista diversifamilia* | HL20100818-01 | S24 | 4 | dead shell | 7.4 | 2.51 | 1.23 | 0.95 | 0.67 | 0.93 | 0.71 | 0.12 | 154.34 | 0.15 | 0.07 | 0.09 | 0.14 | 0.19 | 0.25 |
| HOLOTYPE | NMNS-7276-001 | *Aegista diversifamilia* | HL20100818-01 | S24 | 5 | dead shell | 7.4 | 2.64 | 1.33 | 1.02 | 0.86 | 1.02 | 0.71 | 0.15 | 151.47 | 0.17 | 0.07 | 0.10 | 0.15 | 0.21 | 0.28 |
|  |  | *Aegista diversifamilia* | HL20110915-01 | S25 | 1 | dead shell | 7.1 | 2.41 | 1.18 | 0.90 | 0.73 | 0.96 | 0.64 | 0.11 | 152.27 | 0.18 | 0.07 | 0.10 | 0.14 | 0.18 | 0.23 |
|  |  | *Aegista diversifamilia* | HL20110915-01 | S25 | 2 | dead shell | 6.75 | 2.12 | 1.03 | 0.85 | 0.61 | 1.15 | 0.57 | 0.09 | 152.27 | 0.17 | 0.07 | 0.11 | 0.14 | 0.19 | 0.25 |
|  |  | *Aegista diversifamilia* | HL20110915-01 | S25 | 3 | dead shell | 7.6 | 2.49 | 1.22 | 0.92 | 0.63 | 0.95 | 0.67 | 0.10 | 154.14 | 0.15 | 0.07 | 0.09 | 0.13 | 0.19 | 0.22 |
|  |  | *Aegista diversifamilia* | HL20110915-01 | S25 | 4 | dead shell | 7.4 | 2.33 | 1.12 | 0.88 | 0.61 | 1.59 | 0.61 | 0.11 | 150.79 | 0.14 | 0.06 | 0.10 | 0.12 | 0.16 | 0.24 |
|  |  | *Aegista diversifamilia* | HL20110915-01 | S25 | 5 | dead shell | 7.2 | 2.25 | 1.14 | 0.82 | 0.58 | 0.87 | 0.61 | 0.11 | 150.18 | 0.19 | 0.07 | 0.09 | 0.13 | 0.19 | 0.22 |
|  |  | *Aegista diversifamilia* | HL20110915-01 | S25 | 6 | dead shell | 7 | 2.36 | 1.02 | 0.88 | 0.69 | 0.95 | 0.63 | 0.09 | 161.84 | 0.15 | 0.07 | 0.10 | 0.14 | 0.18 | 0.26 |
|  |  | *Aegista diversifamilia* | HL20100818-04 | S26 | 1 | dead shell | 7.5 | 2.18 | 1.17 | 0.87 | 0.56 | 0.84 | 0.57 | 0.15 | 151.84 | 0.13 | 0.07 | 0.08 | 0.13 | 0.18 | 0.21 |
|  |  | *Aegista diversifamilia* | HL20100818-04 | S26 | 2 | dead shell | 7.15 | 1.98 | 0.97 | 0.78 | 0.56 | 0.90 | 0.53 | 0.08 | 153.10 | 0.13 | 0.06 | 0.09 | 0.11 | 0.16 | 0.21 |
|  |  | *Aegista diversifamilia* | HL20100818-04 | S26 | 3 | dead shell | 7.15 | 2.01 | 1.08 | 0.78 | 0.48 | 1.07 | 0.53 | 0.14 | 149.27 | 0.15 | 0.08 | 0.08 | 0.11 | 0.15 | 0.19 |
|  |  | *Aegista diversifamilia* | HL20100818-04 | S26 | 4 | dead shell | 6.8 | 2.04 | 0.97 | 0.83 | 0.63 | 0.77 | 0.54 | 0.09 | 151.76 | 0.15 | 0.06 | 0.10 | 0.10 | 0.20 | 0.24 |
|  |  | *Aegista diversifamilia* | HL20100818-04 | S26 | 5 | dead shell | 7.15 | 2.12 | 1.02 | 0.80 | 0.64 | 0.84 | 0.55 | 0.08 | 151.49 | 0.13 | 0.07 | 0.09 | 0.13 | 0.17 | 0.21 |
|  |  | *Aegista diversifamilia* | HL20100818-04 | S26 | 6 | dead shell | 7 | 2.17 | 0.99 | 0.89 | 0.67 | 0.77 | 0.63 | 0.08 | 163.12 | 0.14 | 0.06 | 0.09 | 0.13 | 0.19 | 0.22 |
|  |  | *Aegista subchinensis* | YL20110823-02 | S1 | 1 | dead shell | 7.4 | 2.07 | 1.20 | 0.80 | 0.51 | 0.71 | 0.63 | 0.13 | 144.08 | 0.15 | 0.07 | 0.08 | 0.13 | 0.17 | 0.22 |
|  |  | *Aegista subchinensis* | YL20110823-02 | S1 | 2 | dead shell | 7.25 | 1.93 | 1.06 | 0.74 | 0.55 | 0.71 | 0.59 | 0.11 | 148.80 | 0.14 | 0.06 | 0.09 | 0.11 | 0.16 | 0.18 |
|  |  | *Aegista subchinensis* | TP20100109-04 | S2 | 1 | dead shell | 7.2 | 1.93 | 1.02 | 0.73 | 0.43 | 0.68 | 0.56 | 0.11 | 149.28 | 0.16 | 0.05 | 0.09 | 0.12 | 0.16 | 0.19 |
|  |  | *Aegista subchinensis* | TP20100109-04 | S2 | 2 | dead shell | 7.1 | 2.03 | 1.04 | 0.78 | 0.57 | 0.78 | 0.58 | 0.08 | 159.44 | 0.14 | 0.06 | 0.09 | 0.12 | 0.17 | 0.21 |
|  |  | *Aegista subchinensis* | TP20100109-04 | S2 | 3 | dead shell | 7.2 | 1.99 | 1.08 | 0.76 | 0.47 | 0.71 | 0.58 | 0.10 | 146.69 | 0.15 | 0.07 | 0.08 | 0.13 | 0.17 | 0.19 |
|  |  | *Aegista subchinensis* | TP20110515 | S3 | 1 | dead shell | 7 | 2.12 | 1.05 | 0.80 | 0.66 | 0.74 | 0.63 | 0.07 | 155.33 | 0.14 | 0.08 | 0.09 | 0.13 | 0.18 | 0.24 |
|  |  | *Aegista subchinensis* | TP20120424 | S3 | 1 | dead shell | 6.9 | 1.79 | 0.96 | 0.71 | 0.47 | 0.62 | 0.49 | 0.10 | 149.45 | 0.15 | 0.05 | 0.08 | 0.12 | 0.16 | 0.19 |
|  |  | *Aegista subchinensis* | TP20110515 | S3 | 2 | dead shell | 7.6 | 2.24 | 1.18 | 0.84 | 0.60 | 0.84 | 0.65 | 0.10 | 149.19 | 0.15 | 0.06 | 0.09 | 0.12 | 0.15 | 0.21 |
|  |  | *Aegista subchinensis* | TP20120424 | S3 | 2 | dead shell | 7.1 | 1.99 | 1.06 | 0.77 | 0.54 | 0.73 | 0.55 | 0.13 | 151.57 | 0.16 | 0.07 | 0.08 | 0.13 | 0.15 | 0.22 |
|  |  | *Aegista subchinensis* | TP20120424 | S3 | 3 | dead shell | 6.75 | 1.97 | 0.96 | 0.74 | 0.56 | 0.73 | 0.53 | 0.09 | 157.61 | 0.14 | 0.07 | 0.08 | 0.14 | 0.17 | 0.24 |
|  |  | *Aegista subchinensis* | TY20120304 | S4 | 1 | dead shell | 7.25 | 1.93 | 1.10 | 0.75 | 0.45 | 0.66 | 0.58 | 0.14 | 148.57 | 0.13 | 0.07 | 0.08 | 0.12 | 0.16 | 0.20 |
|  |  | *Aegista subchinensis* | TY20120710-02 | S5 | 1 | dead shell | 7.4 | 2.32 | 1.15 | 0.89 | 0.62 | 0.88 | 0.66 | 0.11 | 150.13 | 0.16 | 0.06 | 0.09 | 0.13 | 0.16 | 0.24 |
|  |  | *Aegista subchinensis* | TY20120710-02 | S5 | 2 | dead shell | 7.25 | 1.93 | 1.06 | 0.78 | 0.53 | 0.66 | 0.55 | 0.11 | 147.95 | 0.14 | 0.06 | 0.08 | 0.11 | 0.15 | 0.20 |
|  |  | *Aegista subchinensis* | TY20120710-02 | S5 | 3 | dead shell | 7.7 | 1.97 | 1.14 | 0.78 | 0.49 | 0.70 | 0.57 | 0.14 | 146.38 | 0.12 | 0.06 | 0.08 | 0.11 | 0.15 | 0.18 |
|  |  | *Aegista subchinensis* | TY20120710-02 | S5 | 4 | dead shell | 7.6 | 2.07 | 1.18 | 0.80 | 0.59 | 0.73 | 0.62 | 0.12 | 145.57 | 0.14 | 0.05 | 0.09 | 0.11 | 0.16 | 0.19 |
|  |  | *Aegista subchinensis* | TY20120710-02 | S5 | 5 | dead shell | 7.6 | 2.07 | 1.14 | 0.82 | 0.62 | 0.78 | 0.63 | 0.10 | 147.34 | 0.13 | 0.05 | 0.08 | 0.11 | 0.14 | 0.19 |
|  |  | *Aegista subchinensis* | TY20120710-03 | S6 | 1 | dead shell | 7.25 | 2.11 | 1.15 | 0.83 | 0.55 | 0.77 | 0.61 | 0.12 | 149.58 | 0.16 | 0.06 | 0.09 | 0.12 | 0.18 | 0.19 |
|  |  | *Aegista subchinensis* | HC20101214-03 | S7 | 1 | dead shell | 7.2 | 1.90 | 0.98 | 0.73 | 0.48 | 0.73 | 0.53 | 0.11 | 153.19 | 0.13 | 0.05 | 0.03 | 0.17 | 0.17 | 0.20 |
|  |  | *Aegista subchinensis* | HC20101214-03 | S7 | 2 | dead shell | 7.1 | 1.82 | 0.97 | 0.67 | 0.51 | 0.73 | 0.55 | 0.10 | 153.99 | 0.12 | 0.05 | 0.08 | 0.11 | 0.17 | 0.21 |
|  |  | *Aegista subchinensis* | HC20101214-03 | S7 | 3 | dead shell | 7.1 | 1.91 | 0.98 | 0.71 | 0.50 | 0.71 | 0.56 | 0.08 | 155.33 | 0.13 | 0.06 | 0.08 | 0.12 | 0.15 | 0.23 |
|  |  | *Aegista subchinensis* | HC20101214-03 | S7 | 4 | dead shell | 7.25 | 2.00 | 1.08 | 0.79 | 0.54 | 0.74 | 0.58 | 0.10 | 145.96 | 0.16 | 0.06 | 0.08 | 0.12 | 0.17 | 0.18 |
|  |  | *Aegista subchinensis* | HC20101214-03 | S7 | 5 | dead shell | 7.2 | 1.99 | 1.09 | 0.79 | 0.58 | 0.76 | 0.57 | 0.11 | 149.20 | 0.14 | 0.06 | 0.09 | 0.11 | 0.16 | 0.20 |
|  |  | *Aegista subchinensis* | HC20101214-01 | S8 | 1 | dead shell | 7.2 | 2.02 | 1.09 | 0.79 | 0.52 | 0.75 | 0.60 | 0.10 | 152.40 | 0.16 | 0.06 | 0.08 | 0.12 | 0.18 | 0.21 |
|  |  | *Aegista subchinensis* | HC20101214-02 | S9 | 1 | dead shell | 7.2 | 1.93 | 1.08 | 0.75 | 0.50 | 0.68 | 0.60 | 0.10 | 152.25 | 0.14 | 0.06 | 0.08 | 0.12 | 0.15 | 0.20 |
|  |  | *Aegista subchinensis* | ML20101214-04 | S10 | 1 | dead shell | 7.1 | 1.80 | 0.92 | 0.69 | 0.49 | 0.68 | 0.50 | 0.12 | 150.80 | 0.13 | 0.06 | 0.07 | 0.11 | 0.15 | 0.19 |
|  |  | *Aegista subchinensis* | ML20101214-04 | S10 | 2 | dead shell | 7.2 | 1.79 | 1.03 | 0.71 | 0.43 | 0.61 | 0.54 | 0.10 | 143.02 | 0.14 | 0.06 | 0.07 | 0.12 | 0.13 | 0.19 |
|  |  | *Aegista subchinensis* | ML20101214-04 | S10 | 3 | dead shell | 7.2 | 1.96 | 1.07 | 0.74 | 0.55 | 0.71 | 0.58 | 0.10 | 148.33 | 0.13 | 0.06 | 0.10 | 0.11 | 0.16 | 0.22 |
|  |  | *Aegista subchinensis* | ML20101214-04 | S10 | 4 | dead shell | 7.1 | 1.89 | 0.96 | 0.72 | 0.53 | 0.69 | 0.57 | 0.08 | 152.62 | 0.14 | 0.06 | 0.08 | 0.11 | 0.16 | 0.21 |
|  |  | *Aegista subchinensis* | ML20101214-04 | S10 | 5 | dead shell | 7.7 | 2.06 | 1.14 | 0.78 | 0.56 | 0.74 | 0.59 | 0.14 | 149.49 | 0.13 | 0.05 | 0.08 | 0.13 | 0.15 | 0.18 |
|  |  | *Aegista subchinensis* | ML20101214-01 | S11 | 1 | dead shell | 6.9 | 1.86 | 0.83 | 0.70 | 0.49 | 0.71 | 0.51 | 0.07 | 157.58 | 0.14 | 0.06 | 0.07 | 0.12 | 0.16 | 0.22 |
|  |  | *Aegista subchinensis* | ML20101214-01 | S11 | 2 | dead shell | 7.1 | 1.84 | 0.89 | 0.71 | 0.48 | 0.71 | 0.52 | 0.07 | 156.23 | 0.12 | 0.05 | 0.08 | 0.11 | 0.15 | 0.20 |
|  |  | *Aegista subchinensis* | ML20090118-08 | S12 | 1 | dead shell | 6.85 | 1.97 | 0.98 | 0.70 | 0.51 | 0.76 | 0.55 | 0.09 | 152.16 | 0.17 | 0.06 | 0.09 | 0.13 | 0.18 | 0.21 |
|  |  | *Aegista subchinensis* | ML20090118-08 | S12 | 2 | dead shell | 7.1 | 1.78 | 0.94 | 0.69 | 0.48 | 0.67 | 0.53 | 0.09 | 150.59 | 0.14 | 0.05 | 0.08 | 0.11 | 0.13 | 0.22 |
|  |  | *Aegista subchinensis* | ML20090118-08 | S12 | 4 | dead shell | 7.25 | 2.08 | 1.05 | 0.78 | 0.57 | 0.77 | 0.58 | 0.11 | 151.00 | 0.14 | 0.06 | 0.09 | 0.12 | 0.16 | 0.19 |
|  |  | *Aegista subchinensis* | TC20100122-02 | S13 | 1 | dead shell | 7.2 | 1.91 | 0.97 | 0.71 | 0.45 | 0.71 | 0.54 | 0.10 | 153.61 | 0.14 | 0.05 | 0.09 | 0.12 | 0.15 | 0.18 |
|  |  | *Aegista subchinensis* | TC20100427-02 | S15 | 1 | EtOH | 7.2 | 1.82 | 1.02 | 0.69 | 0.56 | 0.70 | 0.56 | 0.11 | 154.37 | 0.13 | 0.06 | 0.07 | 0.12 | 0.15 | 0.17 |

Sampling site refer to Table 1; Whorl: number of whorl; SW: shell width; SH: shell height; AW: aperture width; AH: aperture height; UW: umbilicus width; BH: body whorl height; SBH: secondary body whorl height; AA: angle of apex; FW: first whorl width; 2W: 2^nd^ whorl width; 3W: 3^rd^ whorl width; 4W: 4^th^ whorl width; 5W: 5^th^ whorl width; 6W: 6^th^ whorl width.
